# 200 Years of Florence and the challenges of nursing practices management in the COVID-19 pandemic*

**DOI:** 10.1590/1518-8345.4576.3358

**Published:** 2020-09-07

**Authors:** Daniela Savi Geremia, Carine Vendruscolo, Ianka Cristina Celuppi, Edlamar Kátia Adamy, Beatriz Rosana Gonçalves de Oliveira Toso, Jeane Barros de Souza

**Affiliations:** 1 Universidade Federal da Fronteira Sul, Curso de Enfermagem, Chapecó, SC, Brazil.; 2 Universidade do Estado de Santa Catarina, Curso de Enfermagem, Chapecó, SC, Brazil.; 3 Universidade Federal de Santa Catarina, Florianópolis, SC, Brazil.; 4 Scholarship holder at the Conselho Nacional de Desenvolvimento Científico e Tecnológico (CNPq), Brazil.; 5 Universidade Estadual do Oeste do Paraná, Curso de Enfermagem, Cascavel, PR, Brazil.; 6 Scholarship holder at the Fundação Araucária, Brazil.; 7 Universidade Federal da Fronteira Sul, Curso de Enfermagem, Chapecó, SC, Brazil.; 8 Bolsista da Scholarship holder at the Coordenação de Aperfeiçoamento de Pessoal de Nível Superior (CAPES), Brazil.

**Keywords:** Coronavirus Infections, Pandemics, History of Nursing, Public Policy, Unified Health System, Public Health, Infecciones por Coronavirus, Pandemias, Historia de la Enfermería, Política Pública, Sistema Único de Salud, Salud Pública, Infecções por Coronavirus, Pandemias, História da Enfermagem, Política Pública, Sistema Único de Saúde, Saúde Pública

## Abstract

**Objective:**

to analyze the main challenges of nursing in facing Coronavirus Disease-19 under the perspective of nurse managers in the west macro-region of Santa Catarina.

**Method:**

it consists of a qualitative study, whose data collection was done through interviews with nurses who represent the management of health care network in the region. The analysis technique used was the Discourse of the Collective Subject (DCS).

**Results:**

the legacy of Florence Nightingale to contemporary nursing practice; the weaknesses and the technical operational capacity with which nursing faces in the Unified Health System (Sistema Único de Saúde - SUS); the strategies for strengthening the Unified Health System and qualification of nursing practices; and the potentialities identified in the pandemic scenario were the main ideas that emerged. In the bicentennial year of Florence Nightingale, nurses recognize her legacy to public health practice and management. Several variables interfere in professional practice, such as epidemiological aspects, working conditions, and care management in a pandemic.

**Conclusion:**

the pandemic scenario has taken nursing to a position of practical and scientific protagonism as a result of its proactivity and leadership in the search for knowledge based on scientific evidence.

## Abstract (es)

**Objetivo:**

analizar los principales retos de la enfermería para hacer frente a la Enfermedad por Coronavirus-19 desde la perspectiva de los enfermeros administradores de la Macro Región Oeste de Santa Catarina.

**Método:**

este es un estudio cualitativo, con recopilación de datos a través de entrevistas con enfermeros que representan la gestión de la red de atención médica en la región. La técnica de análisis utilizada fue el Discurso del Sujeto Colectivo.

**Resultados:**

El legado de Florence Nightingale para la práctica de enfermería contemporánea; las debilidades y la capacidad técnica y operativa que enfrenta la enfermería en el Sistema Único de Salud; estrategias para fortalecer el Sistema Único de Salud y calificar las prácticas de enfermería; y las potencialidades identificadas en el contexto de la pandemia fueron ideas centrales que surgieron. En el año del bicentenario de Florence Nightingale, los enfermeros reconocen su legado para la práctica y gestión de la salud pública. Existen innumerables variables que interfieren en la práctica profesional, en aspectos epidemiológicos, en las condiciones de trabajo y en el manejo de la atención ante la pandemia.

**Conclusión:**

la situación de pandemia ha elevado a la enfermería a una posición de liderazgo práctico y científico debido a su proactividad y capacidad de liderazgo en la búsqueda de conocimientos basados en evidencias científicas.

## Abstract (pt)

**Objetivo:**

analisar os principais desafios da enfermagem no enfrentamento do *Coronavírus Disease-19* sob a perspectiva de enfermeiros gestores na macrorregião oeste de Santa Catarina.

**Método:**

trata-se de um estudo qualitativo, cuja coleta de dados ocorreu por meio de entrevistas com enfermeiros representantes da gestão da rede de atenção à saúde da região. A técnica de análise utilizada foi o Discurso do Sujeito Coletivo.

**Resultados:**

o legado de Florence Nightingale para a prática da enfermagem contemporânea; as fragilidades e a capacidade técnica e operacional com a qual se depara a enfermagem no Sistema Único de Saúde; as estratégias para o fortalecimento do Sistema Único de Saúde e qualificação das práticas de enfermagem; e as potencialidades identificadas na conjuntura da pandemia foram ideias centrais que emergiram. No ano do bicentenário de Florence Nightingale, os enfermeiros reconhecem seu legado para a prática e gestão em saúde pública. São inúmeras as variáveis que interferem na prática profissional, nos aspectos epidemiológicos, nas condições de trabalho e na gestão do cuidado frente à pandemia.

**Conclusão:**

a situação de pandemia tem elevado a enfermagem a uma posição de protagonismo prático e científico pela proatividade e capacidade de liderança na busca de saberes fundamentados em evidências científicas.

## Introduction

The development of nursing faces different challenges concerning autonomy, scientificity, and consolidation of the specific knowledge that characterize the profession. In the period prior to the 20^th^ century, the nursing origins were marked by religiosity and charity as hegemonic aspects of the category. It lived for a long time with the shortage of training institutions, and under the influence of medical professionals on the training and performance of the nurse professional^(1)^.

As a consequence of COVID-19 pandemic (Coronavirus Disease 2019) and the serious structural and political crisis of the Unified Health System (Sistema Único de Saúde - SUS), nursing professionals are even more vulnerable and seek support in the legacy of Florence Nightingale. Being an inspiration and research object for health scholars, undoubtedly, the precursor of nursing contributed to the development of health, since she is considered the founder of modern nursing. Born in Florence (Italy), in 1820, Nightingale used statistical information to establish the conduct of nurses under her management, influencing political and governmental action in the persuasion of authorities. Such action was taken in order to reduce mortality rates by adopting hygiene practices during the Crimean War in 1854^(2-3)^.

The legacy of Florence directed the work of the nurse professional to a performance based on technical-scientific, legal, and political protagonism. This is only possible using practices committed to social well-being in the dimensions of care, management, and research/education. The care and management of nursing requires theoretical support and scientific evidence. Thus, the research contributes to the safety in the performance of the practices, without disregarding the subjective dimension involved in the act of caring and/or managing^(4)^.

Currently, the incorporation of clinical evidence to guide the practice mediated by technologies such as protocols and guidelines, even with timid regulation in Brazil, was responsible for greater visibility and autonomy of nursing and, at the same time, created challenges for the nurse in the Health Care Network (HCN). These challenges are due to the lack of training for the most complex skills, which involve requesting tests and prescribing medications, as well as evidence-based practice^(1)^.

In the midst of the historical context and challenges faced by the professional nurse, in December 2019, after cases reported in the city of Wuhan, China, a new virus from the coronavirus family was discovered. Named as Severe Acute Respiratory Syndrome (SARS-Cov-2), it is responsible for COVID-19. The disease has become a serious public health problem worldwide, evolving very quickly and depleting the responsiveness of health systems.

COVID-19 causes respiratory infections, presenting symptoms that vary in intensity (mild, moderate, or severe) and that are usually intensified by comorbidities presented by individuals. In some cases, the disease may manifest itself in a severe and high lethality^(5)^.

At the beginning of 2020, with the disease widespread in several continents, the World Health Organization (WHO) determined the situation of a pandemic. In Brazil, the condition worsens daily with an ascending characteristic and growth in the epidemic curve, which began on March 2, with two confirmed autochthonous cases. On May 5, 2020, cases totalized 114,715^(5)^. SARS-Cov-2 has been presenting a pattern of high transmissibility in some geographic areas of Brazil. This fast growth has increased the suspected cases, without the necessary notification of confirmation, implying a probable Brazilian undersized epidemic curve, which weakens the strategies to contain the pandemic^(6-7)^.

The estimated death rate among patients treated clinically until May 26, 2020, was approximately 2% of the cases, but the actual number remains unknown, given the underreporting and deaths attributed to other causes due to poor testing. On May 5, 2020, in Brazil, lethality was 6.9%^(5)^, of which the elderly population and those with chronic conditions represent the main risk groups^(8-9)^.

The changes caused by COVID-19 have led to interventions that alter people’s daily lives significantly and put health workers at risk. By acting in the front line, in this context, the nursing team has dealt more frequently with records of contamination, illnesses, deaths, suicides, anxiety and panic crises, as well as the worsening of other diseases, which have been increasingly frequent^(10)^.

Based on these events, the debates that involve the capacity of healthcare services are amplified. Among the key points is the availability of health professionals. Thus, the preservation of the physical and mental health of health workers, which pervades working conditions in the care of victims of COVID-19, is essential for adequate care practices, as well as for the maintenance of the available labor force.

Likewise other historical times when epidemics and disasters have affected populations, nurses have put themselves at risk to provide health care. Thereby, they face problems resulting from the lack of Personal Protective Equipment (PPE)^(11-12)^, inadequate infrastructure of health services^(13)^, exposure to SARS-Cov-2, and long working hours. These problems are combined with fatigue, stigmatization, and physical and psychological violence^(10)^.

Up to 15% of health workers can be infected with SARS-Cov-2 in the world. In Brazil, considering the workers, it is estimated that there will be between 122,000 to 365,000 professionals away from work due to infection, illness, and death by the disease^(9-14)^. As nursing is considered an essential workforce, it is urgent to adopt measures that maintain the performance of these professionals in an attempt to restrict the social and economic impact that their absence could cause, constituting SUS conditions for social and labor dynamics^(9)^. Until May 5, 2020, in Santa Catarina, state with 62,775 nursing professionals, being 15,570 nurses^(15)^, 2,623 cases of COVID-19 were reported, with an incidence of 366 cases *per* one million inhabitants^(5)^. In the west macro-region of Santa Catarina, nurses have led this confrontation through initiatives on several fronts, whether in the Regional Health Management, Municipal Departments, Hospitals or other HCN services. At the same time, nurses lead movements in universities, by developing and collaborating for research on COVID-19, for educational and prevention actions for the general population at risk of illness.

The nurses in the west of Santa Catarina stand out for their strong adherence to hospital services and to Primary Health Care (PHC) and, more recently, for their work in undergraduate and graduate teaching. Based on a path marked by pioneering, nurses “have been promoting changes in nursing practice and teaching, provoking transformations and influencing the culture of nursing care in the region” in different contexts^(16)^.

From a historical perspective, the present study is justified by the debate, since Florence Nightingale, whose bicentenary is celebrated in 2020, the role and contributions of the practices developed by nursing under the perspective of nurses who are in charge of the pandemic in the management of different services and coping with major health emergencies.

It is intended to signalize the main challenges in the action against COVID-19. In order to do so, we started from the following research question: what has challenged nursing in the west macro-region of Santa Catarina in coping with COVID-19?

The objective of this research was to analyze the main challenges of nursing in facing Coronavirus Disease-19 in the west macro-region of Santa Catarina under the perspective of nurse managers.

## Method

Analytical study of a qualitative approach that is part of the multicentric research project entitled “Nursing care and management as knowledge in the field of primary care: proposals for good practices”, approved by the Research Ethics Committee, under record No. 2.380.748/2017, Presentation Certificate for Ethical Appreciation, No. 79506717.6.0000.0118.

In order to carry out this research, interviews were conducted with nurses who contributed and participated in the process of coping with COVID-19 in the organization of front lines for screening and guidance, as well as in the organization of field hospitals, services for the diagnosis of suspected cases and attendance of confirmed cases. Professionals holding management positions at the head of State and Municipal Health Departments, Hospitals, and Universities with undergraduate and graduate courses in nursing were also interviewed, and additionally a supervisor from the Regional Nursing Council (RNC). The inclusion of this last participant is justified by the performance in the inspection to fulfill the minimum requirements for the development of the work with the institutions, such as personnel and PPE.

The nurses’ contacts were known to the researchers, as they are reference personnel for nursing in the region. The research included, by intentional choice, nurses in management functions, totalizing 16 professionals. Among them, there were three non-respondents within the requested time and one refusal, resulting in 12 participants.

The scenario was the west macro-region of the state of Santa Catarina. The information was collected through semi-structured interviews. The guiding questions were: What are the lessons learned from historical nursing practice since Florence that can contribute to nursing practices in coping with COVID-19?; What are the strengths and weaknesses regarding the role of nursing in coping with emergency situations (training, skills, legislation)?; What is your perception about the professional preparation of nurses to work in the dimensions of nursing practice (management, clinic, research) in the current context?; How do you analyze the conditions of technical nursing capacity (assistants, technicians, and nurses in management and assistance) in coping with COVID-19?; How do you analyze the conditions of operational capacity (healthcare networks) of SUS in coping with COVID-19?; How do you analyze governmental action (municipal, state, and federal) in guaranteeing the right to health in times of pandemic?; From the situational and health policy conjuncture, what strategies are necessary for strengthening the SUS and for improving nursing practices in the west macro-region of Santa Catarina?

Due to the social restriction caused by COVID-19 pandemic, potential participants were initially consulted through the WhatsApp messaging application.

After the first contact, an email was sent with a link to access a Google Forms form, which contained in its initial structure the acceptance of the Free and Informed Consent Form (FICF), followed by the interview questions.

From the date the forms were sent by e-mail, seven working days were waited for the forms responses. During the period, the project team made up to two contacts with the participants to remind them of the remaining time to answer the questions sent. The information was produced in April 2020. To obtain the data in order to ensure the quality and reliability of the study, the principles of the Consolidated Criteria for Reporting Qualitative Research (COREQ) were followed.

The data were organized and analyzed manually, using the Discourse of the Collective Subject (DCS) technique. In this proposal, we sought to extract from the interviews: 1) Key Expressions (KE), consisting of literal excerpts or transcriptions of the discourse that reveal the essence of the discursive content; 2) Central Ideas (CI), which are statements that translate the essence of the discourse in order to briefly describe its meaning. CI can be redeemed by direct or indirect/mediate descriptions of the meaning of the statement that reveal the subject of the statement^(17-18)^.

The analysis followed the following steps: (1) exhaustive reading of each interview transcript; (2) identification of themes and grouping of KE; (3) identification of major themes; (4) identification and grouping of KE by theme; (5) identification of the CI in each theme; (6) elaboration of the DCS; (7) analysis of the set of DCS in each theme^(17-18)^.

**Figure 1:**
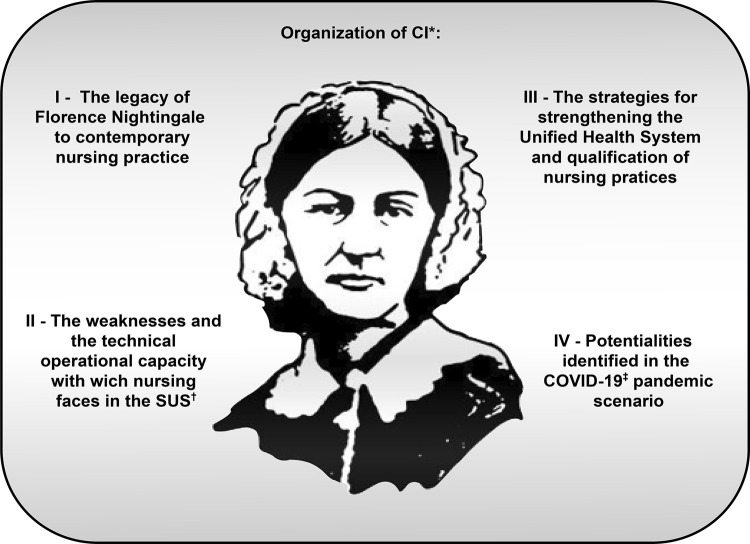
Organization of CI*. West Santa Catarina, 2020 *CI = Central ideas; ^†^SUS = Unified Health System (Sistema Único de Saúde); ^‡^COVID-19 = Coronavirus Disease 2019

Source: Adapted by the authors, from https://br.pinterest.com/pin/336151559683773515/

Representative CI emerged from the challenges mentioned by nurse managers, which were organized as shown in Figure 1. The analysis totalized 27 DCS, which were prepared in the first person singular and listed sequentially.

## Results

Responses were obtained from 11 female participants and one male, who reported having a *lato* or *stricto sensu* postgraduate degree, aged between 34 and 59 years and training time in undergraduate nursing between 10 and 30 years. The time in the SUS management ranged between one to 20 years.

The CI and DCS are presented below. The first CI identified Florence Nightingale’s main legacy for contemporary nursing practice.

*The environmental measures recommended by Florence, such as the practice of hand washing and care with the lighting and ventilation of the environment, represented the initial impulse for the formation of a scientific profession, based on safety care* (DCS 1); *Prophylaxis methods for numerous pathogens and diseases represent much of nursing practice, historically and daily* (DCS 2); *In addition to caring for the environment, Florence contributed to evidence-based studies, with the construction of graphs that allowed the analysis of risk/protection factors in the face of epidemics/pandemics, which contributed to primary health care* (DCS 3); *The constitution of knowledge combined with practice has been fundamental for the restructuring of care, from the time of Florence to the current pandemic condition of COVID-19, in which teams are reinventing themselves and reorganizing themselves to better manage their units* (DCS 4).

We present below the CI that point out the weaknesses of nursing and the technical and operational capacity that nursing faces with in the west macro-region of Santa Catarina.

*In this scenario of coping with the coronavirus pandemic, nursing professionals suffer with low wages, excessive workload, inadequate staff sizing, inappropriate working conditions, and lack of professional recognition* (DCS 5); *In practice, it can be identified weaknesses related to the decision-making by the nurse, leadership and communication, what hinders the understanding and organization of the team. In this context, the importance of managerial development and planning skills by nurses are identified, especially risk management in hospital organizations* (DCS 6); *There is a lack of qualified professionals to take over the new ICU beds opened in the region, whereas recent graduates take time to develop certain skills* (DCS 7); *There are weaknesses related to the compliance with rules and norms, mainly, regarding contact precautions, hand washing, use of PPE, and rationalization of the use of materials* (DCS 8); *Nursing is capable to organize and competently play its role in confronting COVID-19, on both management and clinical levels. In some municipalities, the health care network is articulated to address the pandemic. There was collaborative construction and preparation coordinated by the Municipal Health Departments and Municipal Health Surveillance sectors. New measures are being adopted as required* (DCS 10); *Health services are aligned with the municipality, state and Federal Government in relation to COVID-19. The organization of care for non-serious patients in Health Centers and other points [...] have significantly reduced the care in hospitals* (DCS 11); *Universities have also contributed with telephone consultations with health students, which has been contributing to the distribution of patients in health services according the operational capacity that each municipality has* (DCS 12).

The CI that highlight possible strategies for strengthening SUS and the qualification of nursing practices are presented below.

*Nursing has never been so evident at a time when it is paradoxically advantageous to achieve its deserved visibility. Do we need to go through a serious pandemic to value the biggest category of healthcare professionals? In Brazil, there is no recognition of legal backing for the autonomous and independent performance of nursing in relation to the medical category. No matter how much we talk about protocol approval for nursing practice, we do not advance in a national initiative that enables the autonomy of the profession* (DCS 14); *Nursing should advance in the improvement of its clinic and in the technical, scientific and political empowerment of the category* (DCS 15); *Why have we never seen a nurse as a minister of health? Are there no nursing professionals with this profile or competence? Of course there is! This reveals a lot the so-called and the restriction not yet overcome in the figure of the doctor in relation to health in Brazil* (DCS 16); *Maintaining differentiated funding for health at this time of pandemic is necessary. However, its maintenance beyond this scenario would allow regional transformation* (DCS 17); *Up to the present moment, the west region has shown exemplary in terms of service organization, health responsibility, and planning to tackle the pandemic in the local context* (DCS 18).

There are presented below the potentialities identified in the context of the COVID-19 pandemic, strategies to face the current moment and draw perspectives for the future of SUS and the profession.

*Nursing has a capacity for adaptation and engagement as few professional categories have. We handle adversity very professionally* (DCS 19); *Multi and uni-professional residency program have been very important to deal with the coronavirus. The professionals are acting directly in the service for 60 hours per week. They are experiencing and performing activities that require their theoretical and practical knowledge* (DCS 20); *As leaders of health teams in PHC, nurses can count not only on nursing staff, but with a multidisciplinary health team, incorporating knowledge of several areas to deal with COVID-19* (DCS 21); *Currently, professionals have technologies that permit access to epidemiological information in real time, as well as facilitated access to new legislation. Furthermore, in order to develop skills and also to facilitate the coping with moments of crisis in public health, professional training also increased* (DCS 22); *Regarding the investment of resources, measures of social isolation and availability of materials and PPE were taken* (DCS 23); *Nursing needs to improve its knowledge to ensure patient safety and security. Getting to know new technologies that contribute to this process is fundamental* (DCS 24); *About the technical preparation to face the pandemic, it is noticed that the major centers and capitals have faster access to information, and also to greater planning* (DCS 25); *As Brazil had time to get prepared for the pandemic, from the experiences of several other countries, it managed to organize itself minimally, which also reflected in the work of nursing* (DCS 26); *We are living in a historic moment with great changes for nursing. In teaching, I glimpse a nursing protagonist in the construction of their own knowledge, with more scientific basis. In management, I see a more proactive and leadership-enabled nursing. In care, a notice a nursing that seeks to base its care on best practices and good nursing practices. It is expected that from this pandemic, Brazil will invest more in research, in quality academic training by taking advantage of all its technical capacity* (DCS 27).

## Discussion

By identifying the perceptions that are common to a given group, the DCS method made it possible to understand the moment of the pandemic from the perspective of the nursing managers who represent the west macro-region of Santa Catarina. The data showed dimensions of an action permeated by feelings of dissatisfaction and satisfaction; devaluation and professional recognition; hope and future prospects, revealing sometimes outbursts about the different realities of the region in confronting COVID-19.

The CI identified in the data were discussed based on scientific literature in order to articulate the theoretical aspects that influence illness since Florence to the nursing professional practices in the context of pandemic.

The first CI, which regards the Legacy of Florence Nightingale for the practice of nursing, highlights the importance of hygiene practices over two hundred years. They culminated in innovative techniques and disease prevention measures involving the mobilization of the environment and social backgrounds, intensifying the health promotion of individuals and communities. Thus, by conducting research based on evidence, from notes and records, it was possible to transpose the empirical knowledge to scientific^(3-19)^.

With personal and environmental hygiene during the Crimean War, from 1853 to 1856, Florence managed to reduce mortality by 72% in only eight months, consecrating her a milestone in history as sanitarian and manager^(20)^. From the legacy of the precursor of nursing, the knowledge brought to daily life, such as those with the environment and the washing of the hands, became scientifically grounded.

After 200 years, Brazil and the world resume the most effective actions for the control and prevention of diseases to face COVID-19, reiterating to the medical and scientific communities a consensus on the measures to be implemented in pandemic situations, which are: avoiding agglomerations; performing hand, surface and environmental hygiene; encouraging respiratory hygiene; not sharing personal objects; and finally, detecting and quarantining suspected and confirmed cases^(3,9)^.

Nightingale’s environmental theory provided nursing with a new discipline based on its own body of knowledge mediated, giving the nursing professional support and authority to act freely. By consolidating partnerships to defend the autonomy of nurses, as well as its freedom to act and think during professional activity, the need for a training centered on the assumptions of nursing science has emerged^(20)^.

In this perspective, it was up to the nurses the excellence regarding the comprehensive vision and the commitment as agent of social, organizational, and political changes, being this professional co-responsible for the sustainable development of the nursing work process aiming at individual and collective health^(2)^.

The second CI addresses the Weaknesses and the technical and operational capabilities that nursing faces in the SUS. It is also noteworthy that in the year of the bicentenary of Florence’s birth, the Pan American Health Organization and the World Health Organization defined 2020 as the international year of nursing and obstetrics professionals. However, it was not expected that in this exact year the world would also be surprised by the COVID-19 pandemic. In this scenario, nurses who participated in this study see Florence as an example for those who are working on the frontline in the fight against the pandemic.

In addition, the International Council of Nurses, the WHO, and the UK All Party Parliamentary Group on Global Health launched the Nursing Now Campaign in 2018, which will be completed in 2020. This campaign has the participation of more than 30 countries, including Brazil. The movement aims to enhance nursing, highlighting its importance to improve health services around the world^(21)^.

The current scenario does reflect, however, on the (de)value of nursing. After 200 years, more than half of nursing professionals in Brazil survive on low wages and exhaustive working hours, without realizing the reflection and relevance of their work in society. In order to achieve healthy working conditions and exercise the profession with fullness and security, there is a need to persist in the struggle for better working conditions and for the recognition of the essentiality of nursing in health services^(22)^.

When carrying out a brief historical rescue, it becomes clear that the nursing contingent has expanded significantly in Brazil with the implementation of SUS and the opening of new undergraduate courses. We are witnessing the expansion of research and an expressive growth of clinical case studies, in addition to an increase in professional Masters, Doctors, and Post-Doctors. However, the ideal nursing workday is not regulated by law yet and varies between 30 hours and 40 to 44 hours a week. A heavy workload can negatively affect quality of life, making other activities unfeasible: physical, cultural, which are essential to promote the health of professionals^(22)^.

In the context of combating the COVID-19 pandemic, the lack of hospital beds and equipment, such as mechanical respirators, is one of the problems for work management that impact the health of nurses and other components of the care team^(23)^ in Brazil and abroad^(24)^. In addition, there are failures in the protection of workers, causing contamination, illness, and even the death of some involved in victims’ attendance.

In Brazil, there are 157 nursing professionals who have died of COVID-19 and 3,000 confirmed cases in the category. These numbers placed the country with the highest number of professionals dead and lethality of 5.23% until May 28, 2020^(15)^. Thus, care for health promotion and disease prevention among health workers, which includes nurses, must be prioritized, ensuring access to PPE and training for its correct use^(25)^.

The lethality of a disease cannot be attributed exclusively to the number of intensive care beds available, but this is certainly one of the essential elements to analyze the situation. The lack of PPE and adequate personnel to deal with public health emergencies must also be taken into account. In this pandemic, the need for medium and long-term investment rises in order to adapt the needs to the health care capacity in the health system. In this regard, public policies need to be rethought, especially those aimed at protecting workers, investing in the health system and ensuring the protection of professionals^(8)^.

The third CI concerns strategies for strengthening the SUS and qualifying nursing practices. Despite all the advances that SUS has presented in more than 30 years of its implementation, through processes of political, administrative, and distribution of health services throughout the national territory, the system still faces important weaknesses, worsen by the situation of the pandemic. Among them, we highlight the numerous disassembling actions that the State has implemented in recent years, such as the adoption of strategies to reduce the size of the SUS. The imposed fiscal austerity accentuated the system’s underfunding crisis, impacting directly the functioning, structure, management, and model of the assistance network^(6-18)^.

These health problems that the country accumulates need substantial and continuous investments to overcome the weaknesses in the operational structure of SUS, so that professionals can ensure the integrality and continuity of care in the healthcare network.

Thus, the actions and strategies that could be implemented are: the guarantee of adequate financing for SUS with the repeal of Constitutional Amendment 95/2016, which establishes an expenditure ceiling and a freeze on investments in health until 2036; the definition of career plan, positions and salaries for health workers; the approval of the 30-hour week working journey; the appreciation of PHC; the reorganization of flows with the expansion and qualification of the HCN; greater alignment in arrangements; and agreements between the three federated entities. These are some of the complex elements that can strengthen the SUS and offer better working conditions^(18-26)^.

Nursing constitutes half of the health workforce, and nurses are appointed as the main responsible for coordinating health teams at different levels of care. One of the strategies for investing in the workforce and valuing nurses for the advancement of the profession is the articulation between educational institutions and health services^(21)^. In addition to encouraging the use of research to ensure better nursing practices, the approximation of professionals to real health production scenarios allows the recognition of the potentialities and weaknesses of the system, as well as the needs users have, making them protagonists in this production^(27)^.

Nursing has shown the dimension of its importance in combating COVID-19. Even with the lack of professionals to act in the face of the pandemic and with a context of confrontation, which has at times cost these professionals their lives, the time is ripe for Brazilian nursing to leverage their visibility, demonstrating competence in the face of the current scenario.

Florence’s legacy marked the construction of a body of knowledge proper to modern nursing and the dialogue with other areas of knowledge. With the support given to evidence-based performance and the expansion of functions that, not widely regulated in the country, brought prominence to nurses. A report on the Pan American Health Organization (PAHO) stories page^(28)^ highlights the daily work of two nurses with advanced practices in caring for people with COVID-19. Nevertheless, this autonomy arising from the knowledge acquired in daily practice causes difficulties, especially in PHC^(29)^.

However, the deficit in quality training makes it necessary to adopt strategies that help at work. The support of permanent education, combined with the use of protocols and evidence-based practice, contributes to developing resilience in the nursing workforce^(30)^ and to the profession’s autonomy. It should also be noted that such autonomy is based on the use and incorporation of the nursing process, and in line with its theoretical roots.

The professional nurse needs to seize the methods, processes, and technologies that promote different human functions. It is also advisable to debate and reflect on the training of personnel in order to break with a model that focuses on compliance with hospital prescriptions and routines. It is time for nurses to be valued as a critical, reflective, and autonomous profession^(20)^.

In developed countries, such as England, Spain, the United States, Canada, and Australia, nursing works by developing advanced nursing practices in numerous contexts, but mainly in PHC^(31-33)^. With different levels of autonomy between the mentioned countries, an advanced practice nurse accompanies patients with chronic conditions through the management and handling of cases, including ordering tests and prescribing medications. This practice is guided by care protocols and is supported by the laws of those countries^(33)^.

In times of a pandemic, such as that of COVID-19, clinical nursing care based on knowledge and autonomy^(29)^ reinforces the forefront of health professionals for the care of at-risk groups, which includes people with chronic conditions and the elderly. In this regard, Brazil needs to advance in the discussion about the implementation of advanced nursing practices so that the principles and guidelines that guide SUS are made effective, especially in PHC^(18-29)^.

As presented in the fourth CI, which portrays the potential identified in the context of the pandemic of COVID-19, nursing has a prominent role in SUS. Nurses take the lead in health services, whether with the nursing teams, teams of the Family Health Strategy, with individuals and populations. The necessity of developing environments in which nurses can work to the maximum of their abilities is emphasized, assuming greater influence and responsibility in decision-making on health, social and economic policies^(34)^.

In coping with COVID-19, the insecurity of acting in a new and little-known situation emerges, shaping coping with it according to the experience generated in the work routine itself^(25)^. In this sense, nursing has faced COVID-19 as a team, as it should be, respecting differences and understanding the complementarity of knowledge and practices in the organization of work^(35)^.

Macro and micropolitics determine the health model and way of acting in nursing, as they shape nurses’ practice and workplaces at the local, regional, national, and international levels. In this scenario, nurses commonly act as policy implementers, but they rarely play a central role in their development, assuming a leadership role in the areas of health and social policy^(36-37)^.

In short, it was observed in the discourse of the nurse managers a concern with working conditions in the face of the pandemic and the SUS situation. Despite the weaknesses pointed out by the participants, which include academic training, new professionals with little experience, and the difficulties of performance related to the work process, stood out as potentialities: the importance of single and multi-professional residences; the ability of nursing adapting; the increase in permanent education courses and activities; the new views of society in favor of valuing nursing; the importance of the organization and functioning of services at the three levels of care, as well as their responsiveness in the SUS.

The relationship between the conditions observed for the development of nursing practices and the service capacity will require the resumption of the fundamental pillars of SUS. Such requirements are related to the perspective of structuring and organizing health actions and services in regionalized and hierarchical networks of care, composed by distinct technological densities and improvements in the regulation between the supply and demand for health actions and services^(26)^.

This study has limitations because it analyzes the perception about coping with COVID-19 only under the perspective of nurse managers and does not approach other professionals who are on the front line of the pandemic. It is worth highlighting the importance of studies that address the perspective of professionals who provide care to SUS users. Despite not being the most opportune moment for such investigations, the results of this nature may be important and contribute to the enhancement and recognition of nursing and SUS.

The results of the study contribute to the role of service management for nursing as it explores the difficulties faced and the potential of work management. It also shares the experiences and makes it possible, through scientific knowledge, to reflect on the actions and acquire resilience at work during the COVID-19 pandemic.

## Conclusion

This study brings advances by analyzing the main challenges of nursing performance in SUS in facing COVID-19 in the west macro-region of Santa Catarina under the perspective of managers of different HCN scenarios. The main challenges presented are related to the epidemiological aspects of the high transmissibility, contamination, and deaths caused by coronavirus to the population in general, with emphasis on the high lethality rate of nursing professionals; the poor working conditions, due to the lack of personnel with technical capacity, excessive workloads, low wages and adequate infrastructure; and, still, deficit of financial resources for management and operationalization of the system and the comprehensive care, aggravated by the pandemic of COVID-19.

Thus, the article addressed Florence’s legacy for contemporary nursing practice, the weaknesses and technical and operational capacity of nursing in SUS, strategies for strengthening and qualifying nursing practices, ending with the potentialities identified in care management in the pandemic.

In the year that Florence Nightingale’s bicentenary is celebrated, nurses recognize her legacy and consider it for their public health practices and management. However, it is necessary to move forward in the field of scientific knowledge regarding the performance in pandemic scenarios, understanding the adversities of the profession and its multi and interprofessional relationships in times of crisis in public health.

This historic moment of the pandemic will mark public health as a result of the transformations caused by a virus that has spread rapidly throughout the world. The current scenario has valued nursing for its role in the construction of its body of knowledge, in the proactive organization of care and the SUS, in its leadership capacity, and in the development of knowledge and skills based on scientific evidence, with emerging trends after Coronavirus Disease-19.
